# Dietary Salt-Related Determinants of Hypertension in Rural Northern Thailand

**DOI:** 10.3390/ijerph18020377

**Published:** 2021-01-06

**Authors:** Pimbucha Rusmevichientong, Celina Morales, Gabriela Castorena, Ratana Sapbamrer, Mathuramat Seesen, Penprapa Siviroj

**Affiliations:** 1 Department of Public Health, California State University Fullerton, Fullerton, CA 92831, USA; 2 Department of Health Sciences, California State University Northridge, Los Angeles, CA 91330, USA; celina.morales.285@my.csun.edu; 3 Camp Fire Afterschool Program, Toccoa, CA 92867, USA; gabbyc9614@gmail.com; 4 Department of Community Medicine, Faculty of Medicine, Chiang Mai University, Chiang Mai 50200, Thailand; ratana.sapbamrer@cmu.ac.th (R.S.); mathuramat.s@cmu.ac.th (M.S.); penprapa.s@cmu.ac.th (P.S.)

**Keywords:** hypertension, knowledge, attitude, dietary-salt consumption, consumer behaviors, eating habits

## Abstract

Hypertension and its connection to high salt consumption have been observed in the Thai population. This study mainly contributed to the literature to examine the dietary-salt-related determinants associated with the risk of hypertension in rural northern Thailand, which exhibited the highest prevalence of hypertension. A total of 376 adults residing in San Pa Tong District, Chiang Mai province, were face-to-face interviewed using a structured questionnaire assessing dietary-salt-related knowledge, attitudes, consumption, sources, and habits. The subject’s blood pressure (BP) was measured twice before and after the interview. Hypertension was defined as a systolic BP ≥ 130 mmHg or a diastolic BP ≥ 80 mmHg. The dietary-salt-related knowledge, attitude, and habits toward salt reduction were positively correlated; however, knowledge and attitudes were not significantly correlated with consumption. Multivariate logistic regression results indicated subjects who frequently bought ready-to-eat food, ate out, or used bouillon cube/monosodium glutamate (MSG) during food preparation were likely to have hypertension (OR = 2.24, 95% CI: 1.36–3.69, *p* = 0.002). MSG was heavily consumed and used as a flavor enhancer in northern Thai cuisine; however, a few subjects realized it contains sodium due to no salty taste. The deficiency of specific dietary-salt-related knowledge illustrated the need for tailored educational intervention strategies.

## 1. Introduction

Hypertension, also known as high blood pressure (BP), is a leading global risk factor contributing to cardiovascular diseases, including stroke, coronary artery disease, heart failure, atrial fibrillation, and peripheral vascular diseases [1,2,3]. Specifically, hypertension globally accounts for an estimated 54 percent of all strokes and 47 percent of coronary artery diseases [4]. Hypertension is common in Southeast Asia populations [5,6]. One-third of adults in the Southeast Asian region have hypertension, and nearly 1.5 million deaths are attributed to hypertension every year [7]. In Thailand, about 25% of Thai people have hypertension, and only one-third manage their hypertension [8]. Hypertension is considered the third major risk factor associated with the national disease burden, and it causes 600,000 disability-adjusted life year (DALY) losses per year in Thailand [9,10]. The overall hypertension prevalence among Thai adults increased from 21% in 2003 to 25% in 2014 [9,11,12].

Several aspects of dietary intake are implicated in hypertension’s pathogenesis, including excessive sodium intake [13], low potassium intake, high alcohol consumption, and suboptimal dietary pattern [14,15]. A connection between hypertension and sodium intake has been extensively studied among the Thai population. According to the World Health Organization (WHO) [16], Thai adults consume, on average, 10.8 grams of salt per day, which exceeds the WHO recommended amount of fewer than 5 grams per day. The primary sodium consumption source among Thai people is from salt added during cooking preparation [17].

Although hypertension rates continue to rise in Thailand [9,18] and sodium consumption associated with hypertension has mostly been observed in urban areas [19,20]. The rural areas have shown a lesser prevalence of hypertension than urban areas [21,22,23]. However, the findings were inconclusive. Based on the Thai National Health Examination Survey (Thai NHES IV) in 2014 [12], the prevalence of hypertension was found similar among populations living in urban and rural areas [8]. One cross-sectional study [18] using the same Thai NHES IV from 2008 to 2009 indicated that people living in rural areas had a higher non-compliant risk to daily sodium intake recommendation than people living in urban areas.

By the geographic variations, the northern region (33%) exhibited the highest rate of hypertension, followed by the southern region (28%), the central region (23%), Bangkok (23%), and the northeast region (21%), respectively [8,9]. These statistics are also supported by the median amount of sodium consumption, in which people residing in the northern region of Thailand consumed the most considerable amount (3044.1 mg/day) compared to the other regions and exceeded the daily sodium intake recommendation [18]. Aung et al., 2012 [24], conducted a cluster-randomized trial among high cardiovascular disease risk patients attending diabetes and hypertension clinics at health centers in Muang District, Chiang Rai province, located in the northern region of Thailand. The study suggested that 84% of hypertensive individuals did not know they had hypertension; 73% of prehypertensive individuals did not think they were at risk. About half of all individuals were unaware of the factors associated with hypertension and the impact of salt consumption.

The unawareness of uncontrolled hypertension and the effect of excessive salt consumption is a critical concern in the rural community. Several studies have tried to determine associated factors of hypertension in Thai rural populations [10,25,26]. Only a few studies specifically focused on the rural northern region [24,27]. Deciphering associated factors of hypertension in this population should have been given more attention before implementing hypertension control strategies in this region. This study contributes to the literature to examine the dietary-salt-related determinants associated with the risk of hypertension in the rural northern region, which exhibited the highest prevalence of hypertension in Thailand.

## 2. Materials and Methods

### 2.1. Sample

The data were collected from a cross-sectional survey conducted in Ban Klang Subdistrict of San Pa Tong District in Chiang Mai province, located in the rural northern region of Thailand. A convenience sample of five villages out of eleven villages in the subdistrict was chosen for primary data collection, including (1) Thung Sieo Village (*N* = 1462), (2) Ton Kok Village (*N* = 390), (3) Phra Chao Thong Thip Village (*N* = 429), (4) Pa Sak Village (*N* = 254), and (5) Tong Fai Village (*N* = 722). A minimum sample size (*n* = 356) from the collective five participating villages was calculated using Taro Yamane’s Formula (*n* = *N*/(1 + Ne^2^), *n* = 3257/(1 + (3257 × 0.05^2^) = 356) [28], where *n* is the sample size; *N* is the total target population, and *e* is the margin of error. Individuals who were over the age of 18 years, residing in the participating villages, and capable of providing voluntary informed consent were eligible for inclusion in the study. A total of 376 Thai adults participated in the study.

### 2.2. Procedure

The Thai version of the survey questionnaire was created, then translated back to English using the repeated forward-backward translation procedure to ensure the original meaning was preserved [29]. A survey questionnaire was tested by volunteer college before the data collection in the field. In June 2019, a face-to-face interview was conducted in the Thai language at the respective village community centers by students and faculty from (the institution name is temporarily removed for blind review) and faculty from (the institution name is temporarily removed for blind review). Interviewers read the questions aloud in the Thai language and marked the subjects’ answers. Physical body measurements, i.e., BP, height (cm), and weight (kg), were performed and collected by trained health care professionals in the field. Before the interview, each subject was invited to sit in a comfortable position for having their first BP measurement using a calibrated automatic BP monitor (Omron Blood Pressure Monitor). After completing the 20-minute interview and resting at least 5 min, each subject was measured their BP the second time. Subjects received a participation gift equating to $1.00 (30 THB).

### 2.3. Compliance with Ethical Standards

The research was reviewed and approved by an affiliated Institutional Review Board (the institution name is temporarily removed for blind review) (#HSR-18-19-712, 6/21/19), and subjects provided informed consent. The research complied with the ethical standards as laid down in the 1964 Declaration of Helsinki and its later amendments.

### 2.4. Measures

#### 2.4.1. Hypertension

The study used the BP measurement criteria from the 2017 AHA/ACC guidelines for the prevention, detection, evaluation, and management of high BP in adults [30], which changed the diagnostic threshold and the management goal of BP from 140/90 to 130/80 mmHg for a clinic, home, and daytime measurement. This change in the BP threshold benefited hypertension management in Asian countries in which the BP-dependent characteristics of cardiovascular disease in Asians are stronger than Westerners [31]. A subject’s average systolic BP and average diastolic BP from twice measurements were calculated. Hypertension was a binary outcome variable, taking a value of 1 or 0. If a subject’s average systolic BP was equal or greater than 130 mmHg or their average diastolic BP was equal or greater than 80 mm Hg, the outcome variable was assigned to the value of 1 for having hypertension, otherwise taking a value of 0 for not having hypertension. In Table 1, approximately 70% of the subjects (*n* = 260) in the sample were hypertensive in this study.

#### 2.4.2. Dietary-Salt-Related Knowledge

Subjects were asked a series of nine close-ended questions of dietary-salt-related knowledge. The knowledge questions presented in Table 2 were adapted from the study of Khokhar et al., 2018 [32]. Subjects answered each question in one of these responses: yes, no, or do not know. The first four questions were knowledge about food high in salt, including (1) do shrimp paste and fermented fish contain low salt?, (2) do Monosodium Glutamates (MSGs) contain sodium ingredients though they do not have a salty taste?, (3) do food preservatives contain salt ingredients?, and (4) are chicken bouillon cubes made from chicken and do not contain any salt? The last five questions were knowledge about the effect of salt on health outcome, including (5) does high salt food consumption increase chances of developing hypertension?, (6) does high salt food consumption increase the chances of developing chronic kidney disease?, (7) does high salt food consumption increase chances of developing stroke?, (8) does high salt food consumption increase chances of developing stomach cancer?, and (9) does consuming salt more than 2 teaspoons per day not affect health? The total knowledge scores ranging from 0 to 9 were calculated from the correct answers to the nine questions. The “do not know” response was considered as an incorrect answer. The scores were calculated for overall, non-hypertensive, and hypertensive groups.

#### 2.4.3. Dietary-Salt-Related Attitudes

Subjects were asked four dietary-salt-related attitude questions, each of which was measured on a five-point Likert scale (1 = strongly disagree, 2 = disagree, 3 = neither agree nor disagree, 4 = agree, or 5 = strongly agree). The attitude questions presented in Table 3 were adapted from the study of Khokhar et al., 2018 [32]. The four questions of positive attitudes included (1) you do not believe salt needs to be added extra on food to make it tastier, (2) you think your sodium consumption does not exceed the daily recommended amount, (3) you think it is important to lower dietary salt consumption, and (4) you think there should be laws which limit the amount of salt added to processed foods. The total positive attitude scores ranging from 4 to 20 were calculated for overall, non-hypertensive, and hypertensive groups. The higher scores reflect the positive attitudes toward dietary-salt reduction.

#### 2.4.4. High Dietary-Salt Food Consumption

Subjects were presented a list of eighteen popular Thai food items, which are high in salt and commonly consumed by northern Thais. The list of these food items presented in Table 4 was selected and adapted from the Thailand Food Composition Database [33]. Subjects were consecutively asked whether they ever ate each of these food items in the past three months. If yes, subjects were then asked how many days per week, on average, they ate that food item. The average days per week of all eighteen food items consumed, ranging from 0 days to 7 days a week, were computed for overall, non-hypertensive, and hypertensive groups. The value of 0 means subjects never ate any of these eighteen food items in the past three months. The value of 7 means subjects ate from one of these eighteen food items daily in the past three months.

#### 2.4.5. High Dietary-Salt Food Sources

Food sources that influence high sodium consumption presented in Table 5 included ready-to-eat food, food away from home, and food seasoned during preparation at home [34,35,36]. Subjects were asked whether they ever ate food from each of these sources in the past three months. If yes, they were then asked how many days per week, they ate food from this source? For food prepared at home, subjects were asked whether they ever used bouillon cube or seasoning in food preparation or whether they ever used MSG in the past three months. If yes, subjects were asked how many days per week, on average, they used this seasoning during food preparation at home. The average days per week of food consumed from these sources ranging from 0 days to 7 days a week were calculated for overall, non-hypertensive, and hypertensive groups. The value of 0 means subjects never ate any food from these sources in the past three months. The value of 7 means subjects ate food from one of these sources daily in the past three months.

#### 2.4.6. Dietary-Salt-Related Habits

The habits that restrain from sodium overconsumption presented in Table 6 included tasting food before seasoning, reading the amount of salt on the nutrition label, and not adding additional salt or fish sauce to the food. The habit questions were adapted from the Pan American Health Organization’s salt questionnaire [37] and Kim et al., 2007 [38]. Subjects were asked how often they practiced each of these habits in the past three months on a five-point Likert scale (1 = never, 2 = rarely, 3 = sometimes, 4 = often 5 = always). The total habit scores ranging from 3 to 15 were calculated for overall, non-hypertensive, and hypertensive groups; the higher scores reflect healthier habits toward lower dietary salt intake.

#### 2.4.7. Sociodemographic Variables

The sociodemographic variables were included in the analysis, i.e., gender, elderly, marital status, education, self-reported family history of hypertension, exercise, and Body Mass Index (BMI). An elderly variable was binary, taking a value of 1 if a subject aged 60 years old or up, or otherwise a value of 0 if a subject aged below 60 years old. The education variable was categorized into two levels, taking a value of 1 if a subject had at least junior high school education, or otherwise taking a value of 0 if a subject had lower than junior high school education. A self-reported family history of hypertension variable was assigned a value of 1 if a subject was aware of having at least one family member with high BP, or otherwise taking a value of 0 if a subject was unaware of having any family member with high BP. The measured weight and height of each subject were converted into an indicator of body fatness using a standard BMI formula: weight (kg)/height (m)^2^ [39]. The BMI variable was categorized into three categories: (1) normal and underweight (BMI < 23), (2) overweight (23 ≤ BMI < 27.5), and (3) obese (BMI ≥ 27.5). It is worth noting that the BMI criteria used in this study were based upon a recommended BMI cut-off point for determining overweight and obesity in Asian populations [40].

### 2.5. Statistical Analysis

The univariate analyses were used for sample characteristic description. The bivariate analyses were utilized to identify the sociodemographic and predictor variables associated with hypertension. Specifically, a two-sample *t*-test compared quantitative variables between hypertension and non-hypertension groups. A Pearson’s correlation test determined a relationship between two quantitative variables, and a chi-squared test was for categorical variables associated with hypertension. A multivariate analysis was employed to examine the strength of significant associations between predictor variables and hypertension. In particular, a logistic regression model estimated the dietary-salt-related knowledge, attitudes, habits, and behaviors associated with the risk of hypertension while adjusting for confounding factors, such as sociodemographic variables. The statistical significance level was measured at *p* ≤ 0.05. Data were analyzed using Stata software, version 14 (College Station, TX, USA).

## 3. Results

The sociodemographic characteristics of the subjects are presented in Table 1. About 27% were male, and 73% were female. A majority of the subjects were hypertensive (69.15%), elderly (55.85%), and had an education level of less than a junior high school (72.61%). Two-thirds of the subjects were married (61.70%), and only one-third exercised (31.91%). About 60% of the subjects were overweight or obese. Almost half of the subjects (48.40%) reported having at least one family member with high BP. The distribution of sociodemographic characteristics mostly found significant differences between hypertensive and non-hypertensive groups.

The dietary-salt-related knowledge associated with hypertension is presented in Table 2. Overall, the total knowledge scores were 5.05 ± 1.93. About one-third of the subjects knew that MSGs and food preservatives contain sodium or salt ingredients (Q2, Q3). More than 50% of the subjects had knowledge about high salt food consumption leading to higher chances of developing hypertension (Q5) and diseases such as chronic kidney disease (Q6), stroke (Q7), and stomach cancer (Q8). Specifically, in comparison for specific knowledge about high salt food consumption and the increased chance of hypertension (*Q5*) and developing stroke (*Q7*), the percentage of hypertensive subjects who had these knowledges were significantly higher than the non-hypertensive counterparts (*Q5*: 84.23%, 74.14%; *p* = 0.021; *Q7*: 70.38%, 54.31%; *p* = 0.002).

The dietary-salt-related attitudes associated with hypertension are presented in Table 3. Overall, the total positive attitude scores were 12.72 ± 1.78. On average, subjects neither agreed nor disagreed with positive attitudes. Interestingly, subjects disagreed that they do not believe salt needs to be added extra on food to make it tastier (Q1: 2.82 ± 1.10) and disagreed that their sodium consumption does not exceed the daily recommended amount (Q2: 2.16 ± 0.79). For a specific attitude that believes no need for salt added extra to make food tastier, the hypertensive subjects had significantly higher attitude scores than the non-hypertensive counterparts (2.89 ± 1.22, 2.65 ± 1.05; *p* = 0.042).

The high dietary-salt food consumption associated with hypertension is presented in Table 4. Overall, high dietary-salt food items in which more than 70% of the subjects consumed at least once in the past three months included shrimp paste (93.35%), foods that contain soy sauce (87%), fermented beans (80.05%), fish sauce (78.99%), fermented fish (78.19%), and MSG (77.13%). About one-third of the overall subjects consumed high dietary-salt foods at least five days a week (31.12%). The percentage of hypertensive subjects who consumed these foods at least five days a week was significantly higher than the non-hypertensive counterparts (34.62%, 23.28%; *p* = 0.028). Overall subjects consumed high dietary-salt foods about four days per week (4.04 ± 1.45). For a specific food that contains MSG, hypertensive subjects significantly consumed this food item more often than the non-hypertensive counterparts (5.81 ± 1.96, 5.30 ± 2.13; *p* = 0.050).

The high dietary-salt food sources associated with hypertension are presented in Table 5. Overall, about 66% of the subjects bought ready-to-eat food, and 40% ate food away from home at least once in the past three months. Almost all subjects reported cooking or preparing food at home at least once in the past three months (98.40%). More than 70% of overall subjects reported using bouillon cube or seasoning (73.12%) and using MSG in food preparation (72.85%) at least once in the past three months.

When considering the frequency of consumption within a week, overall subjects bought ready-to-eat food (3.22 ± 2.13) or ate food away from home (2.97 ± 2.06) about three days a week, whereas cooked or prepared food at home almost every day (6.65± 1.14). In addition, subjects used bouillon cube/seasoning (5.42 ± 2.09) or MSG (5.90 ± 1.88) in food preparation more than five days a week. Subjects with hypertension consumed high dietary-salt foods from these sources significantly more often than those without hypertension (5.79 ± 1.35, 5.32 ± 1.35; *p* = 0.002). Specifically, subjects with hypertension used MSG during home food preparation significantly more often than subjects without hypertension (6.12 ± 1.72, 5.42 ± 2.11; *p* = 0.005). Approximately 70% of the overall subjects reported consuming foods from these sources at least five days per week. The percentage of hypertensive subjects who consumed food from these foods at least five days per week was significantly higher than the non-hypertensive counterparts (74.62%, 65.52%; *p* = 0.050).

The dietary-salt-related habits associated with hypertension are presented in Table 6. Overall, the dietary-salt-related habit scores were 9.77 ± 2.71. More than 50% of the overall subjects often/always taste food before seasoning, read the amount of salt on the nutrition label, and not add salt or fish sauce to the food (55.3%). However, there was no difference in habit scores between hypertensive and non-hypertensive subjects.

The correlation coefficients among dietary-salt-related knowledge, attitudes, food consumption, food sources, and habits are presented in Table 7. The dietary-salt-related knowledge was positively correlated with positive attitudes (*r* = 0.144; *p* = 0.005) and with salt reduction habits (*r* = 0.249; *p* < 0.001). The positive attitudes were positively correlated with salt reduction habits (*r* = 0.117; *p* = 0.024). The frequent consumption of high dietary-salt food was positively correlated with the frequent consumption from high dietary-salt food sources (r = 0.267; *p* < 0.001) and negatively correlated with salt reduction habits (*r* = −1.60; *p* = 0.002). The frequent consumption from high dietary-salt food sources was negatively correlated with salt reduction habits (*r* = −1.109; *p* = 0.034).

The multivariate logistic regression results are reported in Table 8. Results were presented in adjusted odds ratios (aORs) with significance levels and 95% confidence intervals (CI). The aORs were reported in this study, describing an association between hypertension and characteristics measured simultaneously. The hypertensive group was compared with the non-hypertensive group, which was a reference group. Subjects who often bought ready-to-eat food, ate out, or used bouillon cube/MSG during food preparation at home for at least five days a week were more likely to have hypertension (aOR = 2.56, 95% CI: 1.55–4.24, *p* < 0.001). Subjects who were elderly (aOR = 2.17, 95% CI: 1.26–3.72, *p* = 0.005), married (aOR = 1.84, 95% CI: 1.12–3.02, *p* = 0.015), or had family members with high BP (aOR = 1.90, 95% CI: 1.15–3.15, *p* = 0.010) were more likely to have hypertension. Lastly, subjects who were overweight (aOR = 1.87, 95% CI: 1.10–3.13, *p* = 0.021) or obese (aOR = 4.61, 95% CI: 2.03–10.48, *p* = 0.010) were more likely to have hypertension.

## 4. Discussion

This study provides insights regarding the dietary-salt-related determinants associated with the risk of hypertension in rural northern Thailand. Similar studies from other countries showed that dietary-salt-related knowledge, attitudes, and consumption are associated with hypertension [41,42,43]; however, this study found only frequent consumption from high dietary-salt food sources was significantly associated with hypertension. Major risk factors such as elderly and body weight were associated with a higher risk of hypertension. The findings supported the high prevalence of hypertension among the elderly as the risk increases with age [16]. Lifestyle modifications, i.e., less dietary-salt consumption and weight control, have been shown to decrease hypertension [44].

Dietary-salt-related knowledge and positive attitudes were not significantly associated with the risk of hypertension. However, hypertensive individuals descriptively presented higher knowledge and attitude scores than non-hypertensive individuals. These knowledge and attitudes may be gained from required regular doctor visits among hypertensive patients [45,46,47]. It is worth noting that even though knowledge and attitudes can evoke efforts to reduce dietary-salt consumption, this study found that they were not significantly correlated with consumption. Another study conducted in Thailand among hypertensive patients in Bangkok showed that although hypertensive patients were aware of their uncontrolled hypertension, they still chose to have a high salt diet due to a preference for tastier and instant food [48]. The pattern of the findings from other countries was similarly observed, such as in Ethiopia [49,50], Nepal [42], India [51,52], Australia [53], Iran [54,55], Spain [56], and Korea [57]. These studies revealed that individuals generally have a reasonable knowledge of the salt and its adverse effects of overconsumption as well as their favorable attitudes toward salt reduction; however, almost none of them took action to reduce salt intake, and their blood pressure was still uncontrolled.

The frequent consumption of high dietary-salt food from these sources, i.e., ready-to-eat food, food away from home, home food cooked with a bouillon cube or MSG, was found positively associated with hypertension. Hurried lifestyles influence ready-to-eat or semi-cooked food [58]. Ready-to-eat food in Thailand includes a wide variety of commonly consumed dishes such as noodles, soups, vegetables, and meat dishes, which are typically purchased from modern food retailers such as stalls, vehicles, and food shops, and restaurants [59]. Access to these modern food retailers has increased due to urban migration and encroachment of these retailers into rural parts of Thailand [60]. The association between ready-to-eat food and hypertension was observed in one study among Thai open university students who regularly purchased ready-to-eat or instant food [16]. It is noted that these food sources often contain high amounts of MSG. For specific high dietary-salt food items and sources in Table 4 and Table 5, hypertensive individuals consumed foods that contain MSG and used MSG in food preparation significantly more often than non-hypertensive individuals. The findings are also supported by the study of Shi et al., 2011 [61], which indicated that the amount of MSG consumption was significantly higher among people in the hypertensive group than in the non-hypertensive group.

MSG is a food additive widely used as a food enhancer in Thai cuisine, especially in northern food. A study of hypertension among the hill tribe population in rural northern Thailand [62] revealed that more than 40% of hill tribe individuals typically used a high amount of MSG when cooking food. Insawang et al., 2012 [63] estimated Thai adults, on average, consume MSG 4.0 ± 2.2 g/day (range from 0.4 to 14.0), which is considered excessive consumption. The higher amounts of MSG consumption are associated with the risk of metabolic syndrome, and hypertension is a classical feature of metabolic anomalies [63]. Approximately one-third of hypertensive patients were reported to have metabolic syndrome [64,65].

In the dietary-salt-related knowledge question asking whether the subjects know that MSGs contain sodium ingredients though they do not have a salty taste, only one-third of them answered this question correctly. No significant difference in this knowledge between hypertensive and non-hypertensive individuals was indicated. Although sodium content in MSG (12.28 g/100 g) is about 60% less than in dietary salt (34.94 g/100 g) [66], consumers may overuse MSG and increase sodium intake because MSG is less salty than the salt of equivalent sodium content [67].

In conclusion, this study found that even though overall dietary-salt-related knowledge and attitudes are associated with habits, they are not translated to the frequency of food consumption and food sources. One implication for public health educational promotion among rural northern Thais should take cultural sensitivity into consideration, such as local food preference, traditional cooking methods, and daily habits that may lead to high dietary salt intake. Conventional education may not be suitable for elderly Thais residing in rural areas with low literacy. Individualized counseling and home visits may increase adherence to hypertension management and prevention [68,69]. Family supports for their hypertensive members, such as preparing foods with low sodium, encouraging them long-term lifestyle modification, monitoring their food consumption, regularly reminding them to take blood pressure medication, can also reinforce hypertension control [70]. Some elderly Thais may eat low-cost food away from home or ready-to-eat foods because of affordability, limited availability of healthy foods, and convenience [71]. Local health professionals should assess the rationale of eating food away from home and ready-to-eat foods, when providing nutritional food information.

### Limitations

Given most elderly in the sample, age-related factors may affect the ability to obtain valid information about food intakes, such as hearing, vision, and memory loss [72], particularly for questions based upon subjects’ recall ability. Thus, there may be response biases in the frequency of food consumption and food source recall. Furthermore, even though the frequency of high dietary-salt food consumption was evaluated, the amount consumed (portion sizes) each time per day and per week was not considered in the questionnaire. The omission of separate portion size questions in favor of a simplified food frequency questionnaire (FFQ) can be an advantage to reduce the respondents’ cognitive burden and to increase data completeness, especially in large-scale epidemiologic studies [73], among those with low literacy or those who prepare a substantial portion of their food at home [74]. Lastly, this is a cross-sectional study; further longitudinal research is needed to observe the causal effect of dietary-salt-related determinants on hypertension risk and the temporal relationship between them.

## 5. Conclusions

Hypertension is common and on the rise among the Thai population. To the best of our knowledge, there is no study examining the dietary salt-related determinants associated with the risk of hypertension among the Thai adult population residing in rural northern Thailand. Dietary-salt-related knowledge was positively correlated with positive attitudes toward dietary-salt reduction; however, these knowledge and attitudes were not associated with consumption. Monosodium Glutamate (MSG) was found to be widely and heavily used as a flavor enhancer in northern Thai cuisine; however, only a few subjects knew that MSG contains sodium despite no salty taste. The deficiency of specific dietary-salt-related knowledge illustrated the need for individualized educational intervention strategies. Primary care units and village health volunteers can play an essential role in educating people in rural communities to reduce salt consumption effectively.

## Figures and Tables

**Table 1:** Sociodemographic characteristics of subjects associated with hypertension.

Demographics	Overall	NHG ^a^	HG ^b^	*p*-Value
**Gender***n* (%)				
Male	102 (27.13)	25 (21.55)	77 (29.62)	0.100 ^c^
Female	274 (72.87)	91 (78.45)	183 (70.38)	
**Age** (Mean ± SD)	59.69 ± 11.71	56.96 ± 12.35	60.91 ± 11.22	0.002 ^d,^**
**Elderly** (y ≥ 60) *n* (%)				
years ≥ 60	210 (55.85)	52 (44.83)	158 (60.77)	0.004 ^c,^**
years < 60	166 (44.15)	64 (55.17)	102 (39.23)	
**Married***n* (%)				
No	144 (38.30)	57 (49.14)	87 (33.46)	0.004 ^c,^**
Yes	232 (61.70)	59 (50.86)	173 (66.54)	
**Education***n* (%)				
Junior high school level or above	103 (27.39)	38 (32.76)	65 (25.00)	0.119 ^c^
Less than junior high school	273 (72.61)	78 (67.24)	195 (75.00)	
**Exercise***n* (%)				
No exercise	256 (68.09)	72 (62.07)	184 (70.77)	0.095 ^c^
Regular exercise	120 (31.91)	44 (37.93)	76 (29.23)	
**High Blood Pressure in Family History***n* (%)				
No	194 (51.60)	72 (62.07)	122 (46.92)	0.007 ^c,^**
Yes	182 (48.40)	44 (37.93)	138 (53.08)	
**Body Mass Index (BMI)**^e^ (Mean ± SD)	24.32 ± 4.60	23.29 ± 3.68	25.05 ± 5.04	<0.001 ^d,^**
Underweight/Normal (BMI < 23) *n* (%)	147 (39.10)	60 (51.72)	87 (33.46)	<0.001 ^c,^**
Overweight (23 ≤ BMI < 27.5) *n* (%)	157 (41.75)	46 (39.66)	111 (42.69)	
Obese (BMI ≥ 27.5) ^e^ *n* (%)	72 (19.15)	10 (8.62)	62 (23.85)	
**Total Observation**	376	116 (30.85)	260 (69.15)	

^a^ NHG (Non-Hypertensive Group): Systolic BP < 130 and Diastolic BP < 80. ^b^ HG (Hypertensive Group): Systolic BP ≥ 130 or Diastolic BP ≥ 80. ^c^ Chi-square test for differences between NHG and HG ^d^ Two-sample *t*-test for differences between NHG and HG ^e^ The BMI criteria used in this study was based on a recommended BMI cut-off point for determining overweight and obesity in Asian populations (WHO Expert Consultation, 2004); ** statistically significant for *p* ≤ 0.01.

**Table 2:** Dietary-salt-related knowledge associated with hypertension.

Questions	Overall (*N* = 376)	NHG ^a^ (*n* = 116)	HG ^b^ (*n* = 260)	*p*-Value ^c^
**Number of subjects who answered the question correctly***n* (%)				
Q1. Do shrimp paste and fermented fish contain low salt?	154 (40.96)	47 (40.52)	107 (41.15)	0.908
Q2. Do MSGs contain sodium ingredients though they do not have a salty taste? ^e^	118 (31.38)	33 (28.45)	85 (32.69)	0.413
Q3. Do food preservatives contain salt ingredients?	104 (27.66)	31 (26.72)	73 (28.08)	0.787
Q4. Are chicken bouillon cubes made from chicken and do not contain any salt?	228 (60.64)	75 (64.66)	153 (58.85)	0.287
Q5. Does high salt food consumption increase the chances of developing hypertension?	305 (81.12)	86 (74.14)	219 (84.23)	0.021 *
Q6. Does high salt food consumption increase the chances of developing chronic kidney disease?	362 (96.28)	113(97.41)	249 (95.77)	0.437
Q7.Does high salt food consumption increase the chances of developing a stroke?	246 (65.43)	63 (54.31)	183 (70.38)	0.002 **
Q8.Does high salt food consumption increase the chances of developing stomach cancer?	207 (55.05)	63 (54.31)	144 (55.38)	0.847
Q9.Does consuming salt more than 2 teaspoons per day not affect health?	175 (46.54)	52 (44.83)	123 (47.31)	0.656
**Total knowledge score (9 points)** (Mean ± SD)	5.05 ± 1.93	4.85 ± 1.87	5.14 ± 1.97	0.188 ^d^

^a^ NHG (Non-Hypertensive Group): Systolic BP < 130 and Diastolic BP < 80. ^b^ HG (Hypertensive Group): Systolic BP ≥ 130 or Diastolic BP ≥ 80. ^c^ Chi-square test for differences between NHG and HG ^d^ Two-sample *t*-test for differences between NHG and HG ^e^ The interviewers explained to the subjects how “sodium” and “salt” are related. * statistically significant for *p* ≤ 0.05; ** statistically significant for *p* ≤ 0.01.

**Table 3:** Dietary-salt-related attitudes associated with hypertension.

Positive Attitude Questions	Overall (*N* = 376) Mean ± SD	NHG ^a^ (*n* = 116) Mean ± SD	HG ^b^ (*n* = 260) Mean ± SD	*p*-Value ^c^
Q1. You *do not* believe salt needs to be added extra on food to make it tastier.	2.82 ± 1.10	2.65 ± 1.05	2.89 ± 1.22	0.042 *
Q2. You think your sodium consumption *does not* exceed the daily recommended amount.	2.16 ± 0.79	2.10 ± 0.85	2.18 ± 0.78	0.341
Q3. You think it is important to lower dietary salt consumption.	3.97 ± 0.86	3.97 ± 0.89	3.96 ± 0.85	0.959
Q4. You think there should be laws which limit the amount of salt added to processed foods.	3.77 ± 0.92	3.78 ± 0.85	3.77 ± 0.95	0.919
**Total positive attitude scores (20 points)** (Mean ± SD)	12.72 ± 1.78	12.50 ± 1.70	12.82 ± 1.82	0.100

^a^ NHG (Non-Hypertensive Group): Systolic BP < 130 and Diastolic BP < 80. ^b^ HG (Hypertensive Group): Systolic BP ≥ 130 or Diastolic BP ≥ 80. ^c^ Two-sample *t*-test for differences between NHG and HG * statistically significant for *p* ≤ 0.05.

**Table 4:** High dietary-salt food consumption associated with hypertension.

High Salt Food Consumption	*n* (%) of Subjects Consumed High-Salt Foods in the Past Three Months				Average Days per Week (Mean ± SD)			
	Overall (*N* = 376)	NHG ^a^ (*n* = 116)	HG ^b^ (*n* = 260)	*p*-Value ^c^	Overall	NHG ^a^	HG ^b^	*p*-Value ^d^
1. Shrimp paste	351 (93.35)	112 (96.55)	239 (91.92)	0.096	5.00 ± 2.17	4.72 ± 2.21	5.13 ± 2.14	0.100
2. Food that contains soy sauce	328 (87.23)	100 (86.21)	228 (87.69)	0.690	3.66 ± 2.03	3.59 ± 1.98	3.69 ± 2.06	0.712
3. Food contains fish sauce	297 (78.99)	90 (77.59)	207 (79.62)	0.656	4.19 ± 2.35	4.13 ± 2.31	4.21 ± 2.38	0.791
4. Fermented Bean	301 (80.05)	99 (85.34)	202 (77.69)	0.086	3.99 ± 2.24	4.13 ± 2.28	3.93 ± 2.23	0.519
5. Fermented Fish	294 (78.19)	94 (81.03)	200 (76.92)	0.373	4.88 ± 2.29	4.65 ± 2.28	4.98 ± 2.29	0.284
6. Food that contains MSG	290 (77.13)	90 (77.59)	200 (76.92)	0.888	5.65 ± 2.02	5.30 ± 2.13	5.81 ± 1.96	0.050 *
7. Food that contains oyster sauce	266 (70.74)	87 (75.00)	179 (68.85)	0.226	2.96 ± 1.76	3.05 ± 1.63	2.92 ± 1.83	0.642
8. Thai Sausage	221 (58.78)	66 (56.90)	155 (59.62)	0.621	1.55 ± 1.10	1.50 ± 0.86	1.58 ± 1.24	0.797
9. Canned Fish	207 (55.05)	71 (61.21)	136 (52.31)	0.100	1.39 ± 0.84	1.30 ± 0.76	1.47 ± 0.89	0.490
10. Bean Paste	189 (50.27)	57 (49.14)	132 (50.77)	0.770	1.67 ± 1.29	1.85 ± 1.46	1.57 ± 1.20	0.447
11. Salted Fish	175 (46.54)	57 (49.14)	118 (45.38)	0.500 **	1.86 ± 1.55	1.91 ± 1.68	1.83 ± 1.52	0.882
12. Fruits with chili and salt	173 (46.01)	64 (55.17)	109 (41.92)	0.017 **	2.80 ± 1.88	2.45 ± 1.78	3.05 ± 1.93	0.122
13. Instant Noodle	168 (44.68)	53 (45.69)	115 (44.23)	0.793	1.78 ± 1.22	1.88 ± 1.20	1.74 ± 1.23	0.709
14. Pickles	167 (44.41)	50 (43.10)	117 (45.00)	0.732	2.29 ± 2.09	2.50 ± 2.09	3.24± 2.38	0.240
15. Packaged snacks, i.e., chips	137 (36.44)	40 (34.48)	97 (37.31)	0.599	3.14 ± 2.20	3.76 ± 2.15	2.79 ± 2.18	0.050 *
16. Dried Shrimp	120 (32.00)	39 (33.62)	81 (31.27)	0.653	2.20 ± 1.58	2.00 ± 1.66	2.33 ± 1.55	0.513
17. Salted Eggs	89 (23.67)	29 (25.00)	60 (23.08)	0.685	1.79 ± 1.44	2.14 ± 2.19	1.58± 0.79	0.429
18. Rice Porridge	59 (15.69)	20 (17.24)	39 (15.0)	0.581	2.00 ± 1.03	2.00 ± 1.07	2.00 ± 1.05	1.000
Average days a week on high dietary-salt food consumption	-	-	-		4.04 ± 1.45	3.85 ± 1.50	4.13 ±1.42	0.090
Average at least five days a week on high dietary-salt food consumption	117 (31.12)	27 (23.28)	90 (34.62)	0.028 *				

^a^ NHG (Non-Hypertensive Group): Systolic BP < 130 and Diastolic BP < 80. ^b^ HG (Hypertensive Group): Systolic BP ≥ 130 or Diastolic BP ≥ 80. ^c^ Chi-square test for differences between NHG and HG ^d^ Two-sample *t*-test for differences between NHG and HG * statistically significant for *p* ≤ 0.05; ** statistically significant for *p* ≤ 0.01.

**Table 5:** High dietary-salt food sources associated with hypertension status.

Food Sources ^e^	*n* (%) of Subjects Consumed Food from These Sources in the Past THREE Months				Average Days per Week (Mean ± SD)			
	Overall (*N* = 376)	NHG ^a^ (*n* = 116)	HG ^b^ (*n* = 260)	*p*-Value ^c^	Overall	NHG ^a^	HG ^b^	*p*-Value ^d^
1. Buy ready-to-eat food	248 (65.96)	81 (69.83)	167 (64.23)	0.290	3.22 ± 2.13	2.90 ± 1.84	3.42 ± 2.27	0.100
2. Eat food away from home	152 (40.53)	49 (42.24)	103 (39.77)	0.652	2.97 ± 2.06	2.75 ± 2.07	3.13 ± 2.07	0.463
3. Cook or prepare food at home	370 (98.40)	114 (98.28)	256 (98.46)	0.894	6.65 ± 1.14	6.65 ± 1.14	6.65 ± 1.15	0.964
3.1 Use bouillon cube or seasoning in food preparation	272 (73.12)	84 (73.04%)	188 (73.15)	0.983	5.42 ± 2.09	5.22 ± 2.12	5.51 ± 2.07	0.304
3.2 Use MSG in food preparation	271 (72.85)	87 (76.65)	184 (71.60)	0.416	5.90 ± 1.88	5.42 ± 2.11	6.12 ± 1.72	0.005 **
Average days a week on consuming food from these sources	-	-	-		5.64 ± 1.35	5.32 ± 1.35	5.79 ± 1.35	0.002 **

^a^ NHG (Non-Hypertensive Group): Systolic BP < 130 and Diastolic BP < 80. ^b^ HG (Hypertensive Group): Systolic BP ≥ 130 or Diastolic BP ≥ 80. ^c^ Chi-square test for differences between NHG and HG ^d^ Two-sample *t*-test for differences between NHG and HG ^e^ Subjects were asked how often they practiced each of these habits in the past three months on a five-point Likert scale (1 = never, 2 = rarely, 3 = sometimes, 4 = often 5 = always). ** statistically significant for *p* ≤ 0.01.

**Table 6:** Dietary-salt-related habits associated with hypertension.

Questions	Overall (*N* = 376)	NHG ^a^ (*n* = 116)	HG ^b^ (*n* = 260)	*p*-Value ^d^
**Habit score of each question**^e^ (Mean ± SD)				
1. Taste food before seasoning?	3.95 ± 1.50	3.90 ± 1.59	3.97 ± 1.46	0.685
2. Read the amount of salt on the nutrition label?	2.20 ± 1.39	2.37 ± 1.41	2.13 ± 1.38	0.123
3. Not add salt or fish sauce to the food?	3.61 ± 1.37	3.62 ± 1.26	3.61 ± 1.42	0.884
**Total habit scores (out of 15 points)** (Mean ± SD)	9.77 ± 2.71	9.91 ± 2.75	9.71 ± 2.69	0.525
% of subjects who often or always doing these habits *n* (%)	208 (55.30)	69 (59.48)	139 (53.46)	0.278 ^c^

^a^ NHG (Non-Hypertensive Group): Systolic BP < 130 and Diastolic BP < 80. ^b^ HG (Hypertensive Group): Systolic BP ≥ 130 or Diastolic BP ≥ 80. ^c^ Chi-square test for differences between NHG and HG ^d^ Two-sample *t*-test for differences between NHG and HG ^e^ Subjects were asked how often they practiced each of these habits in the past three months on a five-point Likert scale (1 = never, 2 = rarely, 3 = sometimes, 4 = often 5 = always).

**Table 7:** Pearson’s correlation coefficients among dietary salt-related determinants including knowledge, attitudes, food consumption, food sources, and habits.

Pearson’s Correlation Coefficient	Dietary Salt-Related Determinants			
	Knowledge ^a^	Attitudes ^b^	Food Consumption ^c^	Food Sources ^d^
Knowledge				
Attitudes	0.144 **			
Food consumption	−0.050	−0.050		
Food sources	−0.044	−0.029	0.267 **	
Habits ^e^	0.249 **	0.117 *	−0.160 **	−0.109 *

^a^ Dietary-salt-related knowledge: the higher scores reflect the higher knowledge. ^b^ Dietary-salt-related attitude: the higher scores reflect the positive attitudes toward dietary salt reduction. ^c^ High dietary-salt food consumption: the higher frequency reflects more days per week consuming high dietary-salt food. ^d^ High dietary-salt food source: the higher frequency reflects more days per week consuming food from high dietary-salt food sources. ^e^ Dietary-salt-related habits: the higher scores reflect healthier habits toward lower dietary salt intake. * statistically significant for *p* ≤ 0.05; ** statistically significant for *p* ≤ 0.01.

**Table 8:** Multivariate logistic regression for factors associated with hypertension.

Variables	Adjusted Odd Ratio	95% CI	*p*-Value
Dietary-salt-related knowledge	1.13	0.98–1.29	0.076
Dietary-salt-related positive attitudes	1.11	0.96–1.20	0.100
At least five days a week on consuming high dietary-salt food	1.52	0.86–2.55	0.100
At least five days a week on consuming food from high dietary-salt food sources	2.56	1.55–4.24	<0.001 **
Dietary-salt-related habits	0.87	0.51–1.47	0.598
**Gender** (Reference: Female)			
Male	1.64	0.92–2.90	0.092
**Age group** (Reference: Age < 60 years old)			
Elderly: Age ≥ 60	2.17	1.26–3.72	0.005 **
**Marital status** (Reference: Not married)			
Married	1.84	1.12–3.02	0.015 *
**Education level** (Reference: Less than junior high school)			
Junior high school or higher education	0.73	0.40–1.34	0.310
**Exercise frequency** (Reference: none or sometimes exercise)			
Regular exercise	0.65	0.39–1.11	0.100
**Family history of high blood pressure** (Reference: no member)			
Family members having high blood pressure	1.90	1.15–3.15	0.010 **
**Body Mass Index (BMI)** (Reference: normal weight: BMI < 23)			
Overweight: 23 ≤ BMI < 27.5	1.87	1.10–3.13	0.021 *
Obese: BMI ≥ 27.5	4.61	2.03–10.48	0.010 **
Constant	0.04	0.01–0.30	0.002 **
*N* = 376; Wald chi-square (13) = 45.4; Pseudo R-square = 0.17			

* statistically significant for *p* ≤ 0.05; ** statistically significant for *p* ≤ 0.01.
